# Prediction and evaluation of the lipase inhibitory activities of tea polyphenols with 3D-QSAR models

**DOI:** 10.1038/srep34387

**Published:** 2016-10-03

**Authors:** Yi-Fang Li, Yi-Qun Chang, Jie Deng, Wei-Xi Li, Jie Jian, Jia-Suo Gao, Xin Wan, Hao Gao, Hiroshi Kurihara, Ping-Hua Sun, Rong-Rong He

**Affiliations:** 1 Guangdong Province Key Laboratory of Pharmacodynamic Constituents of TCM and New Drugs Research, College of Pharmacy, Jinan University, Guangzhou 510632, China; 2 Anti-stress Health Research Center, College of Pharmacy, Jinan University, Guangzhou 510632, China; 3 Yunnan University of Traditional Chinese Medicine, Kunming 650500, China

## Abstract

The extraordinary hypolipidemic effects of polyphenolic compounds from tea have been confirmed in our previous study. To gain compounds with more potent activities, using the conformations of the most active compound revealed by molecular docking, a 3D-QSAR pancreatic lipase inhibitor model with good predictive ability was established and validated by CoMFA and CoMISA methods. With good statistical significance in CoMFA (*r*^2^_cv_ = 0.622, r^2^ = 0.956, F = 261.463, SEE = 0.096) and CoMISA (*r*^2^_cv_ = 0.631, *r*^2^ = 0.932, F = 75.408, SEE = 0.212) model, we summarized the structure-activity relationship between polyphenolic compounds and pancreatic lipase inhibitory activities and find the bulky substituents in R_2_, R_4_ and R_5_, hydrophilic substituents in R_1_ and electron withdrawing groups in R_2_ are the key factors to enhance the lipase inhibitory activities. Under the guidance of the 3D-QSAR results, (2R,3R,2′R,3′R)-desgalloyloolongtheanin-3,3′-O-digallate (DOTD), a potent lipase inhibitor with an IC50 of 0.08 μg/ml, was obtained from EGCG oxidative polymerization catalyzed by crude polyphenol oxidase. Furthermore, DOTD was found to inhibit lipid absorption in olive oil-loaded rats, which was related with inhibiting the activities of lipase in the intestinal mucosa and contents.

Tea (*Camellia sinensis*) has been widely consumed as healthy beverages in the world^1^. People worldwide have acknowledged that tea possesses many beneficial effects on their health, particularly the prevention of obesity and the improvement of lipid metabolism^2^. Epidemiological data showed that habitual tea consumption can lower the body fat and regulate the plasma lipoprotein constructions^3^,^4^. Our previous clinical studies confirmed that the polyphenolic compounds of tea with extraordinary hypolipidemic effects^5^. Apart from polyphenols, caffeine is also an important component in tea to promote lipid metabolism. However, the side effects of central nervous system limit the use of caffeine’s thermogenic and fat oxidizing properties. Thus, the lipase inhibitory effect of polyphenols has attracted great attention^6^.

Polyphenols of tea are predominantly catechins and tannins^7^. It is also well-known that heat epimerization produces catechin derivatives during processing and extracting such as (−)-epigallocatechin (EGC), (−)-epicatechin-3-gallate (ECG) and (−)-epigallocatechin-3-gallate (EGCG)^8^. It has been reported that these catechins and their derivatives possess antioxidant, antiglycemic, anticancer and antilipidemic properties^7^. Researchers also reported that theaflavins and the polymerized polyphenols can alter lipid metabolism through inhibiting fatty acid synthesis and enhancing fatty acid oxidation^9^. The study of Nakai *et al.* showed theaflavins had stronger inhibitory activities than tea polyphenols. They assumed that the polymerization of flavan-3-ols and/or the galloyl moieties in their structures might be needed for their enhancement of inhibition of pancreatic lipase^10^. However, this hypothesis needs more validation. In our study, the docking and 3D-QSAR models were performed to depict the structure-activity relationship between polyphenolic compounds and pancreatic lipase inhibition activities, which might indicate some key information for optimizing hypolipidemic activity of the compounds. The polyphenolic compounds identified the common core structure of EGCG. The models were of satisfactory statistical significance and predictive ability, and we were intrigued by the QSAR and those polymers which gave excellent lipid lowering effect. Using the conformations of the most active compound revealed by molecular docking, a 3D-QSAR pancreatic lipase inhibitor model with good predictive ability was also established and validated by CoMFA and CoMISA methods to further understand the structure-activity relationship between polyphenolic compounds and pancreatic lipase inhibitory activities. In order to get compounds with better activities, the polymerized polyphenols were catalyzed by crude polyphenol oxidase according to the QSAR results which indicate better activity of EGCG’s dimer with specific orientation.

## Results

### Docking analysis of tea flowers polyphenols

**Figure 1:**
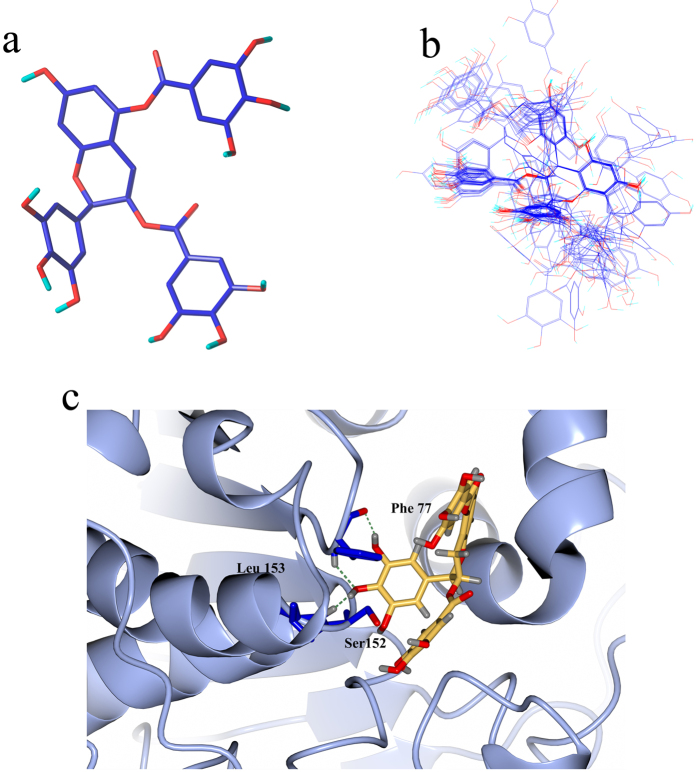
Molecular alignment based on docking results. (**a**) Conformation of compound 8 retrieved from docking result; (**b**) Alignment of the training compounds; (**c**) The binding mode between selected compound 8.

The Fig. 1 depicts the binding modes between the binding pocket of lipase and compound 8, as well as the alignment of compounds based on the conformation retrieved by docking. The docking conformation of template molecule, compound 8, is shown in Fig. 1a. Based on the benzopyrone ring as the common substructure, we can see all molecules overlap well as in Fig. 1b. The hydroxyl groups at pyrogallol in R_1_ position form a H-bond as an acceptor of hydrogen-bond with the -NH group in the Leu153 and Phe77 residues of lipase, while the other two hydroxyls form H-bonds with Ser152 and Phe77 residues to serve as a donor of hydrogen bond. These observations from Fig. 1c precisely match the acceptor contour maps and the donor of hydrogen bond in the corresponding CoMSIA models.

### CoMFA and CoMSIA analysis

**Figure 2:**
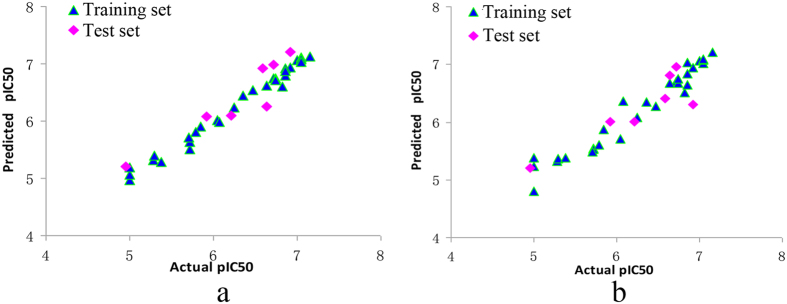
CoMFA and CoMSIA graphs of actual and predicted pIC50 values. (**a**) CoMFA; (**b**) CoMSIA.

Both of the predicted and actual pIC_50_ values of the training and test set compounds are displayed in Table 1. The data from CoMSIA and CoMFA models are depicted graphically as scatter diagrams in Fig. 2a,b, respectively. The two diagrams demonstrate an outstanding match of actual and predicted activities. Table 2 shows the parameters of PLS analysis. The model of CoMFA presents satisfactory leave-one-out cross-validation (*q*^*2*^ value = 0.622), non-cross-validation (*r*^2^ value = 0.956), standard errors of estimate (SEE) of 0.069 and *F* value of 261.463 with an optimal component of 3. The steric field and electrostatic field descriptors explain 44.6% and 55.4% of the variance, respectively. Furthermore, CoMSIA models constitute of descriptors containing steric, hydrophobic and H-bond donors were of a high *q*^*2*^ value (0.631) and *r*^*2*^ value (0.932). Other parameters including the optimal component, SEE and *F* value are 4, 0.122 and 75.408, respectively. Contributions of three selected descriptors are 0.232 for steric, 0.345 for hydrophobic and 0.423 for hydrogen bond donor, respectively.

### External validation

The external validation is a vital part of the model verification and its *r*^2^_pred_ values calculated based on both training and test set are respectively 0.918 and 0.773 for CoMFA and CoMSIA model (Table 2). The favorable external validation parameters indicate that our models are of high accommodating capacities in predicting the pIC_50_ values of new derivatives.

### 3D Contour maps of QSAR

**Figure 3:**
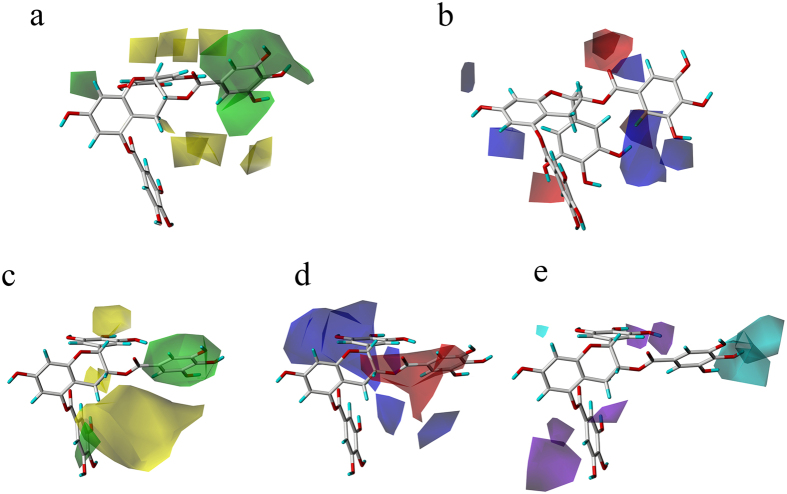
Contours of CoMFA and CoMSIA analysis with compound 8 as template molecule. (**a**) The steric fields of CoMFA. Green contours refer to the regions in which bulky groups elevate activities. Yellow contours suggest the regions in which bulky groups reduce activities; (**b**) The electrostatic fields of CoMFA. The contours in blue and red respectively represent favored regions of electron-donating groups and electron-withdrawing groups; (**c**) The contours in green and yellow respectively indicate bulky and minor groups favored regions; (**d**) Hydrophobic fields of CoMSIA. The contours in blue and red respectively locate the favored hydrophilic and hydrophobic regions; (**e**) Hydrogen-bond donor fields of CoMSIA. The contours in cyan and purple respectively locate the regions in which hydrogen-bond donor groups are favorable and unfavorable.

In this study, compound 8 was used as the template molecule in QSAR models. For CoMFA steric field (Fig. 3a), the green contours (80% contribution) mean these regions are of favorable steric tolerance, and the yellow contours mean 20% contribution for disfavored region. The green contours located around the aromatic ring indicate that this region with bulky substituents would increase the biological activity. That’s why the compounds 3, 5 and 16 where hydroxyl is induced in R_2_ show poorer activity than other compounds with bulky substituents. The red and blue contours in Fig. 3b respectively show the favorable locations for the electronegative and electropositive groups. A red contours encircling the carboxyl of benzoyl ring characterize where positively charged substituents are unfavorable. The higher activities of compounds 1, 2, 4, 8 and 11 than other compounds agree with the conclusion of electrostatic fields map.

As to the CoMSIA contours plots, the steric, hydrophobic, hydrogen bond donor fields are dipicted in Fig. 3c–e. The steric contour maps of CoMSIA are alike to those of CoMFA. In hydrophobic counter maps, red (with 80% contribution) and blue (with 20% contribution) contours indicate hydrophobic and hydrophilic favored sites, respectively. A large blue contour can be observed around the R_1_ position, indicating that hydrophilic substituents induced here can enhance the activity. This confirm that compounds with phenol possessed better bioactivity. The red region near the side chain of R_2_ emphasizes a structural preference of hydrophobic substituents. For instance, compounds 1, 8, 10 and 11 whose substituents in R_2_ are ester groups show better activities. Hydrogen bond donor fields are depicted in purple (20%) and cyan (80%) contours for unfavorable and favorable regions, respectively. Cyan contour located near R_2_ indicates hydrogen bond donor induced here can increase the activity of compounds.

### Structure-activity relationship

**Figure 4:**
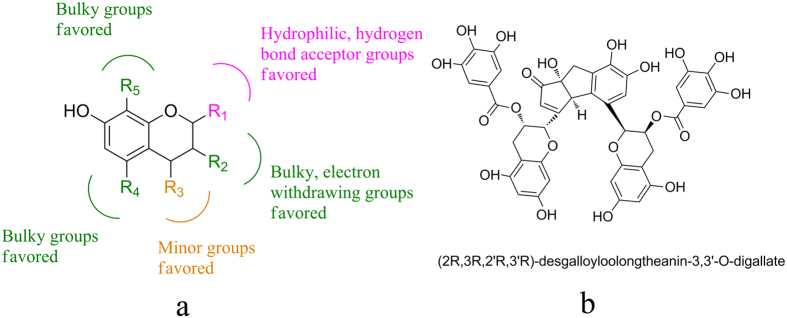
Synthesis guided by SAR. (**a**) The Structure-activity relationship analyzed by 3D-QSAR and docking; (**b**) The Structure of DOTD.

According to the results of docking and 3D-QSAR, the structure-activity relationship of these polyphenols are summarized in Fig. 4a. Briefly, R_1_ site favors the hydrophilic, hydrogen-bond acceptor groups; the bulky, electron withdrawing groups at R_2_ site are likely to improve the activity; the minor and bulky substituent at R_3_ and R_4_ positions would increase the activity, respectively. Bulky groups at R_5_ position are favored to the activity.

### The oxidized tea polyphenol and its inhibitory activity of pancreatic lipase

The structure-activity relationship indicates useful information of rationally designing. Under the guidance of the 3D-QSAR results, a compound named (2R,3R,2′R,3′R)-desgalloyloolongtheanin-3,3′-O-digallate (DOTD) was obtained from EGCG oxidative polymerization catalyzed by crude polyphenol oxidase. The structure of DOTD is shown in Fig. 4b. The effect of DOTD on the pancreatic lipase *in vitro* was determined, and results showed that DOTD showed strong lipase inhibition, with an IC_50_ of 0.08 μg/ml.

### Activities of DOTD on TG level in olive oil-loaded rats

**Figure 5:**
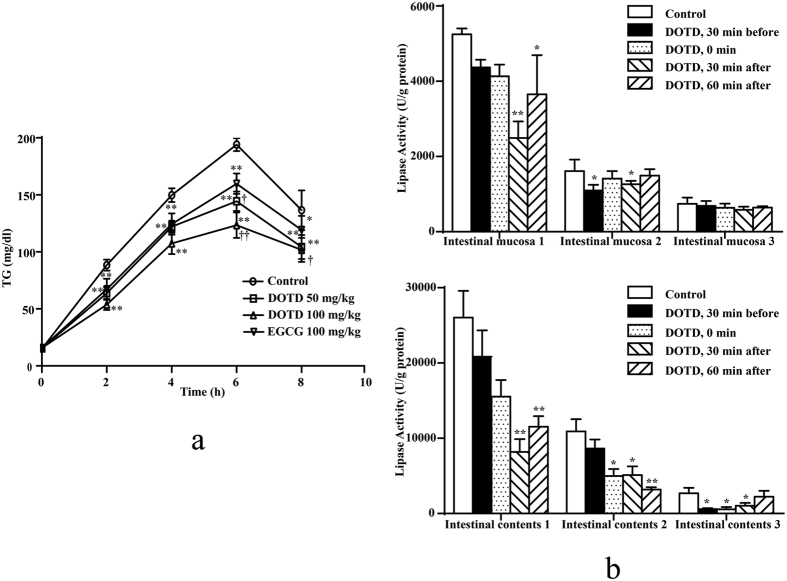
*In vivo* activity of DOTD. (**a**) Effects of DOTD on TG levels in the plasma of rats loaded with olive oil; (**b**) Effects of DOTD on lipase activity in the mucosa and the contents of small intestine of rats loaded with olive oil. Values are expressed as mean ± S.D. (n = 7 in each group). ^*^P < 0.05, ^**^P < 0.01 vs. control group, and ^†^*P* < 0.05, ^††^*P* < 0.01 vs. EGCG group.

Based on the results from molecular docking, we inferred that DOTD might affect lipid absorption by inhibiting pancreatic lipase, thus converts TG to monoglycerides and free fatty acids. Thus, lipid tolerance test was conducted to determine the influence of DOTD on TG level. Figure 5a shows the chronological changes of TG in blood after oral administration of olive oil to rats. In the control group, the TG level in blood reached the peak at 6 h after administration of olive oil. Comparing to the control group, DOTD (50 and 100 mg/kg) significantly decreased the TG level at the time point of 2, 4, 6 and 8 h. DOTD (50 and 100 mg/kg) showed better effects on decreasing TG level in olive oil-loaded rats than EGCG (100 mg/kg) at the point of 6 and 8 h.

### Inhibitory effects of DOTD on lipid absorption in intestine of olive oil-loaded rats

The effects of DOTD on TG level in olive oil-loaded rats might be related with the lipase inhibition. As shown in Fig. 5b, the activities of lipase in both intestine and mucosa contents were obvious higher in intestine section 1 than in section 2 and 3. When DOTD (100 mg/kg) was administrated to rats, the lipase activities were significantly inhibited. These results indicated DOTD with potent lipase inhibitory effect *in vivo*.

## Discussion

In the present study, the developed structure-activity relationships on CoMFA, CoMSIA and docking models emphasize different preference of substituent group in all five substituent position (Fig. 4a). Among these indications, the favor groups in R_1_ and R_2_ position gave us major guidelines in our rational improvement of compounds since most field contours are located around this part. Hydrophilic, hydrogen-bond acceptor groups are favorable at R_1_ position. According to the docking analysis, R_1_ substituent form key interaction with lipase by H-bonding. The pyrogallol here with its multiple hydroxy is more beneficial. The bulky, electron withdrawing groups at R_2_ position are most vital in affecting activity. As can be summarized from both activity data and QSAR contour maps, without bulky, electron withdrawing group like galloyl moieties in R_2_ position, the activity is diminished or even vanished. These conclusions also verify the assumption of Nakai *et al.*^10^.

**Figure 6:**
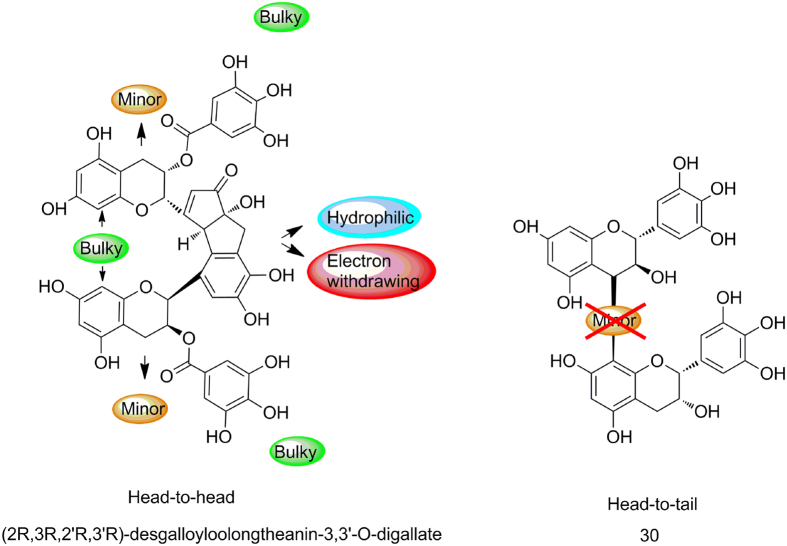
Head-to-head and head-to-tail polymerization.

Along with the information of substituent preference in R_3_ and R_5_ position, we found that the EGCG dimers polymerized in a head-to-head style are of more prominent activity for they precisely match our QSAR results above which indicated bulky, minor, and hydrophilic or electron withdrawing groups in the specific substitute position. As illustrated in Fig. 6, head-to-head polymerization violates no SAR instructions however dimers polymerized in head-to-tail way disobey some inevitably. As to the bulky group preference in R_4_ position, we assumed that galloyl moieties can benefit activity. However, we haven’t obtained a 5-O-gallate dimer yet, this hypothesis needs further verification.

The present research indicated that the beneficial effects of EGCG on obesity and associated pathologies are significantly related to its direct and acute effects on lipid metabolism and energy balance. Evidences we obtained demonstrated that EGCG played a predominant role on dietary lipid metabolism both through stimulating their oxidation and reducing their absorption. In this study, the chemical constituents of tea flower, including petal, stamen and pollen were analyzed. The inhibitory activities of TFE against pancreatic lipase by 37 polyphenols from tea flower were examined *in vitro* and obtained satisfactory results. We also established the docking and 3D-QSAR models of lipase inhibition by CoMSIA and CoMFA methods. Statistical significance was obtained in CoMFA (*q*^2^ = 0.622, *r*^2^ = 0.956, *F* = 261.463, SEE = 0.069) and CoMISA (*q*^2^ = 0.631, *r*^2^ = 0.932, *F* = 75.408, SEE = 0.122) model. DOTD was obtained from EGCG oxidative polymerization catalyzed by crude polyphenol oxidase, and showed a potent lipase inhibitory activity. The content of DOTD in tea flower and tea leaves was comparatively lower, which was difficult to be extracted. Akizawa *et al.* extracted DOTD from oolong and named it oolongtheanin 3′-O-gallate^10^. However, it could be obtained through enzymatic oxidation under a mild condition. This method could be further optimized to allow mass production.

## Materials and Methods

### Chemicals and Regents

C, EGC, EC, ECG, EGCG, CG, GC, GCG and all other compounds used in Table 3 were obtained from Wako Ind. (Osaka, Japan). Olive oil, 4-methylumbelliferyl (4-MU) oleate and type VI-S pancreatic lipase were bought from Sigma-Aldrich (St. Louis, MO, USA).

### Animals

Six-week-old SD male rats were obtained from Guangdong Medical Laboratory Animal Center (Guangzhou, China). All the rats were kept in cages and fed with standard chow and water in animal room with controlled illumination (12 h light/dark cycle), humidity (50 ± 5%) and temperature (23 ± 1 °C). All the animal treatments were performed under the “Guideline for the Care and Use of Laboratory Animals” (No. 85–23, revised 1985), published by National Institutes of Health of U.S. All the animal experimental procedures were approved by the Laboratory Animal Ethics Committee of Jinan University (20120910001).

### Data set

All polyphenols and their bioactivities were showed in Table 4. The demarcation of training and test sets was based on the following criteria as previous report^11^: (I) Molecule diversity was statistically significant. (II) The chosen compounds should be of precise and concise information of activity ranges and structure features to eliminate bias or redundancy. (III) The compounds with most and least activities should be arranged in the training set. With the considerations above, all thirty-seven molecules were assigned randomly with the compounds 6, 11, 14, 15, 21, 31 and 37 chosen as the test set. Table 4 displays the structures of all compounds in the training and test sets. For the convenience in analyzing, the activities of the polyphenols were converted to pIC_50_.

### Molecular docking

Molecular docking is now widely used in studying ligand-protein interaction and new drug discovery^12^,^13^,^14^,^15^,^16^. Many different algorithms were applied in different docking software to explore the affinity of ligands and receptor. In this case, Surflex-Dock in SYBYL 8.1 equipped with an empirical scoring function and a patented search engine was used to dock small-molecules into a protein-active pockets^13^, and applied to investigate the interactions between the compounds and lipase (PDB code: 1LPB)^17^. The idealized hypothetical ligand which makes all potential interactions with the binding site was generated as the protomol with the threshold of 0.5 and bloat of 1 Å, and other parameters are default. Surflex-Dock uses CH_4_, N-H, and C=O fragments as probes to detect into the cavity and gather the information of steric effects and hydrogen-bond sites in the receptor pocket^18^,^19^. Before docking, we repaired the side chain, deleted all the water molecules in the crystal structure, then all hydrogen atoms and Gasteiger charges were added^20^,^21^. A staged minimization was conducted to optimize the receptor. Using the conditions optimized above, compound 8 was docked into the binding pocket. The total scores are expressed as the logarithm of dissociation constant (lgKd) to characterize binding affinities. Ten conformers with highest scores from docking results were ranked in a spreadsheet file, and we took the configuration with highest total score into further investigation in protein-ligand interaction. The CCP4mg program was applied to depict the interactions between compound 8 and lipase graphically^22^,^23^.

### Molecular modeling and alignment

**Figure 7:**
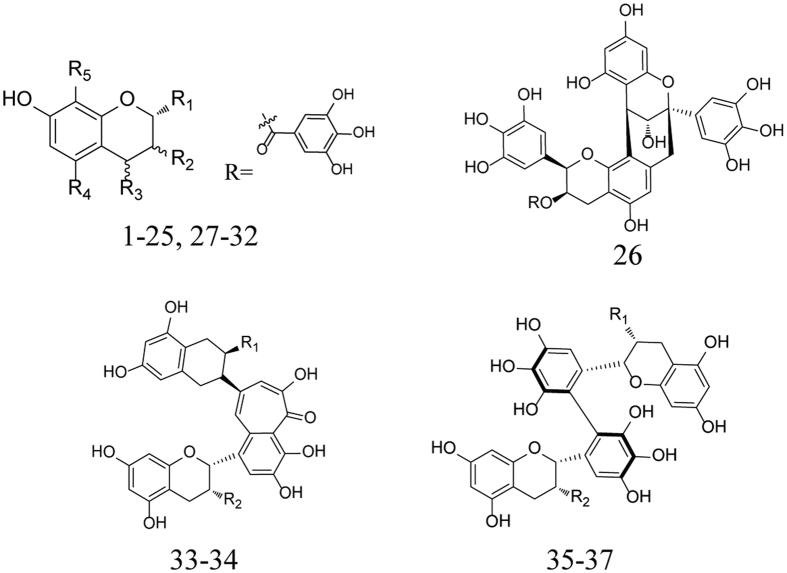
The basic skeletons of the training and test set compounds in Table 3 . Each structure represents the skeleton of the corresponding molecules in Table 3.

All 37 polyphenols’ structures shown in Fig. 7 and Table 3 were prepared in sketch module of SYBYL 8.1 (Tripos, Inc., St. Louis, MO, USA). After the construction of molecules, structural energy minimization was conducted by conjugate gradient methods under the conditions of a gradient convergence of 0.05 kcal/(Å mol) in Gasteiger-Hückel charge. The maximum steps of minimization were set to 20000. Alignment is the most essential procedure in building a reliable model. The predictive ability and the statistic quality of 3D-QSAR models are closely related to the alignment^24^. Normally, three different aligning methods such as pharmacophore, common substructure and docking overlaps are used to obtain convinced alignment^25^,^26^. The compound 8 retrieved from the docking result with the highest active value and reasonable energy (16.205 kcals/mol) was used as the template molecule with benzopyrone ring as the common substructure for its core position and rigid structure.

### CoMFA and CoMSIA setup

CoMSIA and CoMFA are widely used computational methods in 3D-QSAR modeling which could help us to understand the structures of polyphenolic compounds and guide rational structure modification to obtain novel compounds with more potent activity. With the 3D grid spacing of 2 Å in x, y and z axis, the Coulomb potentials and the Lennard-Jones were applied to calculate the energies of steric and electrostatic field^27^. The field values of CoMFA model were detected by a probe atom of a sp^3^ hybridized carbon with the value cut off set at 30 kcal/mol^28^,^29^. For the CoMSIA method, two additional different fields of hydrophobic, hydrogen-bond acceptor were also calculated as an extension of steric and electrostatic fields^30^. In CoMSIA method, similarity indices were introduced to make all grid points calculable. The similarity indices were calculated by equation (1):


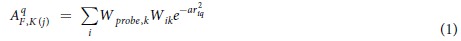


where *q* is the grid point, and *k* indicates the physicochemical properties of electrostatic and steric descriptors; *W*_*probe,k*_ represents the probe atom and the attenuation factor is a default value of 0.3; *i* means summation index calculating on all atoms of the molecule *j*, and *W*_*ik*_ represents the actual value of physicochemical property *k* of the atom *i*^31^.

### Partial least squares (PLS) analysis

The PLS analysis was applied to linearly correlate CoMSIA and CoMFA fields to pIC_50_ values. For this analysis^32^, “leave-one-out” was applied in calculating the cross-validation coefficient (*q*^*2*^) to investigate the robust of the training set. The *q*^2^ is a good indicator of the model fitting and in general, and the *q*^*2*^ value above 0.5 means an acceptable model. Whereafter, the correlation coefficient (*r*^*2*^) was calculated by a non-cross-validation procedure, and the optimized model of 3D-QSAR was generated by using the optimum number of components determined in “leave-one-out”. SEE was calculated by equation (2) to test the model.


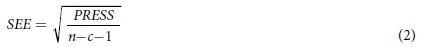


where, n represents the compounds quantity; c represents the counts of components; PRESS means the sum of squared deviations between the observed and predicted values of test compounds.

### External validation

The coefficient *q*^2^ is often identified as a benchmark in verifying the model, however it’s not always adequate. A model with high *q*^2^ and *r*^2^ values doesn’t meant to be absolutely accurate. Even though a model predict the test set precisely enough, the model might perform poorly in predicting when it was applied to another new set of molecules^33^. Therefore, a new parameter of external test coefficient (*r*^*2*^_*pred*_) was introduced in assessing the model’s predictive ability^34^. Predictive values *r*^*2*^_*pred*_ were calculated by the equation (3):


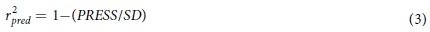


where, SD represents the sum of squared deviations between the activities of test set and training set.

### Preparation of oxidized polymerization from tea polyphenols

The leaves of green tea (*C. sinensis*) were collected in April from tea plants which grow in the mountain in Longmen County, Guangdong Province, China. Fresh tea leaves (300 g) of *C. sinensis* were homogenized in a 1000 mL acetone under cooling. The insoluble components were removal by filtration with gauze, and the remaining extract were freeze-dried and stored in −80 °C freezer. Five grams of above extract was suspended in cysteine phosphate buffer (50 mM, pH 6.0) by stirring for 2 h under 5 °C. The solution was centrifuged (12, 000 rpm, 30 min), and the supernatant was treated with 1.0 M ammonium sulfate. The solution was purified with Sephacryl S-300 and Phenyl Sepharose CL-4B. The targeted fraction was dissolved in citric acid–potassium phosphate buffer (0.01 M: 0.02 M, 600 mL, pH 5.6), and applied to the polyphenol oxidase reaction. EGCG (600 mg) was added to the solution of polyphenol oxidase and incubated for 3 h at 32 °C. Then, 90% of CH_3_CN containing 1% TFA (600 mL) was added to stop this reaction. The reaction product was then diluted 5 times with distilled water and eluted on an HP-20 column. After washing with distilled water, 90% CH_3_CN containing 0.1% TFA was used to elute catechins. Three oxidized polyphenols were isolated and purified by prep-HPLC. The structures of the oxidized compounds were identified by MS and NMR analyses as DOTD (18 mg), prodelphinidin B2 (5 mg) and prodelphinidin A2-3′-O-gallate (4 mg). The experiments above were repeated to obtain enough compounds for further studies. MS and NMR data of DOTD were supplied in the Supporting Information.

### Inhibition of pancreatic lipase *in vitro*

As a substrate, 4-MU oleate was used to test the activity of pancreatic lipase. A mixture of 4-MU oleate solution (50 μL) and different concentrations of test sample (25 μL) were added in the 96-well microtiter plate. Then, lipase solution (50 U/mL, 25 μL) was added and incubated (30 min, 25 °C). To stop the reaction, sodium citrate solution (0.1 M, 0.1 mL, pH 4.2) was added. The level of the product 4-methylumbelliferone was measured by a fluorescence reader (Tecan, Switzerland, Ex/Em = 355 nm/460 nm). The lipase inhibition rate (%) of each test was calculated by equation (4):


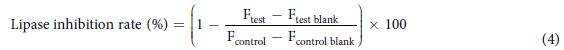


where, F_test_ and F_test blank_ respectively represent the fluorescence values of test samples with and without the substrate 4-MU oleate. F_control_ and F_control blank_ respectively represent the fluorescence values of control with and without the substrate 4-MU oleate.

IC_50_ of the test samples was calculated on the basis of least-squares regression line of plots of different concentrations (log) against the lipase inhibition rate (%).

### Plasma lipid tolerance test

The influence of DOTD on lipid absorption was determined by plasma lipid tolerance test as we previously described^35^. Before this experiment, SD rats were starved for 18 h. DOTD (50, 100 mg/kg) was orally administrated to to rats. Thirty min later, olive oil solution was administered orally at a dosage of 5 mL/kg. Rats in control group received the same volume of distilled water. The chronological changes of blood TG levels in rats were analyzed in rats at time point of 0, 2, 4, 6 and 8 h (n = 7 in each group). Blood samples were distilled from an arterial cannula implanted in the abdominal aorta through the femoral artery of rats under anesthesia. The blood was contained in a tube with sodium heparin (2%) and centrifuged (5,000 rpm, 5 min). The supernatants were used to determine plasma TG level by a commercial kit obtained from Nanjing Jiancheng Bioengineering Ltd. (Nanjing, China).

### Lipase activity in intestinal mucosa

Before this experiment, rats were starved for 18 h. Rats were orally loaded with olive oil solution at a dosage of 5 mL/kg. DOTD (100 mg/kg) was administered to rats at 0 or 30 min before olive oil load, or at 30 or 60 min after olive oil load. All the rats were anesthetized 2 h after olive oil administration. The small intestines were taken out and cut into 3 sections. The intestinal digesta and mucosa were obtained and suspended as 5% PBS solution in tubes and centrifuged (10,000 rpm, 10 min)^36^. The supernatants were collected to determine the lipase activities as described above. The protein level was determined by Coomassie brilliant blue protein quantitative kit obtained from Nanjing Jiancheng Bioengineering Institute (Jiangsu, China).

### Statistical analysis

Docking and QSAR statistical results were summarizing together in Sybyl MDE module^37^,^38^. Data are expressed as means ± S.D. and analyzed by one-way ANOVA followed by Tukey’s *post hoc* test (SPSS Inc, USA). Differences at *P* < 0.05 are considered as significance.

## Additional Information

**How to cite this article**: Li, Y.-F. *et al.* Prediction and evaluation of the lipase inhibitory activities of tea polyphenols with 3D-QSAR models. *Sci. Rep.* **6**, 34387; doi: 10.1038/srep34387 (2016).

## Supplementary Material

Supplementary Information

## Figures and Tables

**Table 1:** Comparison of actual and predicted biological activity in terms of pIC50 of the compounds by using CoMSIA and CoMFA models.

Compound	Actual	CoMFA		CoMSIA	
		Pred.	Res	Pred.	Res
1	6.8861	6.758	−0.1281	6.899	0.141
2	6.6778	6.752	0.0742	6.64	−0.112
3	4.5229	4.821	0.2981	4.749	−0.072
4	6.4685	6.201	−0.2675	6.153	−0.048
5	4.5229	4.891	0.3681	4.921	0.03
6*	6.2441	6.123	−0.1211	6.411	0.288
7	6.4089	6.335	−0.0739	6.33	−0.005
8	6.9586	6.771	−0.1876	7.003	0.232
9	6.1308	6.237	0.1062	5.89	−0.347
10	6.0132	6.062	0.0488	5.964	−0.098
11^*^	6.7959	6.211	−0.5849	6.312	0.101
12	5.9101	5.578	−0.3321	5.563	−0.015
13	6.0315	6.732	0.7005	6.542	−0.19
14*	6.1612	6.09	−0.0712	6.002	−0.088
15*	5.5513	6.085	0.5337	6.001	−0.084
16	5.0605	5.225	0.1645	5.093	−0.132
17	6.7212	6.544	−0.1772	6.523	−0.021
18	5.4841	5.154	−0.3301	5.341	0.187
19	6.6021	6.66	0.0579	6.614	−0.046
20	6.9586	7.05	0.0914	7.059	0.009
21*	6.5229	6.456	−0.0669	6.816	0.36
22	5.6402	5.597	−0.0432	5.674	0.077
23	5.058	5.066	0.008	5.122	0.056
24	5.4841	5.19	−0.2941	5.295	0.105
25	4.7622	5.112	0.3498	5.178	0.066
26	6.7447	6.765	0.0203	6.944	0.179
27	5.4895	5.126	−0.3635	5.272	0.146
28	5.6655	5.579	−0.0865	5.481	−0.098
29	7	6.983	−0.017	6.971	−0.012
30	5.1643	5.196	0.0317	5.171	−0.025
31*	6.6021	6.977	0.3749	6.959	−0.018
32	6.2147	6.396	0.1813	6.076	−0.32
33	6.8861	6.805	−0.0811	6.934	0.129
34	7	6.861	−0.139	7.047	0.186
35	6.8239	6.768	−0.0559	6.802	0.034
36	6.5229	6.599	0.0761	6.565	−0.034
37*	4.7857	5.214	0.4283	5.204	−0.01

^*^Test set.

**Table 2:** PLS statistics of the CoMFA and CoMISA 3D-QSAR models.

PLS	CoMFA	CoMSIA
*q*^2^	0.622	0.631
*r*^2^	0.956	0.932
ONC	3	4
SEE	0.069	0.122
F values	261.463	75.408
Steric	0.446	0.232
Electrostatic	0.554	
Hydrophobic		0.345
H-bond donor		0.423
*r*^2^_pred_	0.918	0.773

*q*^2^: Cross-validated correlation coefficient. *r*^2^: Non-cross-validated coefficient. ONC: Optimal number of components. SEE: Standard error of estimate. F: F-test value. *r*^2^_pred_: Predictive correlation coefficient.

**Table 3:**
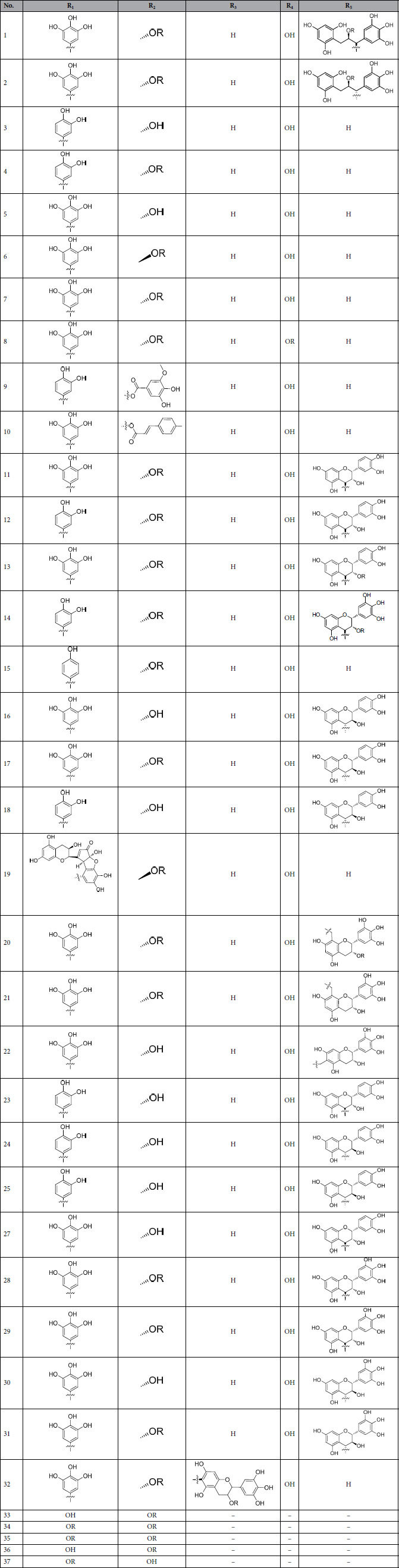
The training and test set compounds.

The basic skeletons of these compounds are shown in Fig. 7.

**Table 4:** Inhibitory Activities of Tea Polyphenols on Pancreatic Lipase.

No	Compoud Name	IC_50_(μM)	pIC_50_
1	assamicain A	0.13	6.8861
2	assamicain B	0.21	6.6778
3	(−)-epicatechin (EC)	>30	4.5229
4	(−)-epicatechin-3-O-gallate (ECG)	0.34	6.4685
5	(−)-epigallocatechin (EGC)	>30	4.5229
6	(−)-gallocatechin-3-O-gallate (GCG)	0.57	6.2441
7	(−)-epigallocatechin-3-O-gallate (EGCG)	0.39	6.4089
8	(−)-epigallocatechin-3,5-di-O-gallate	0.11	6.9586
9	(−)-epicatechin 3-O-(3′-O-methyl)-gallat	0.74	6.1308
10	(−)-epigallocatechin-3-O-p-coumaroate	0.97	6.0132
11	(−)-epicatechin(4β-8)-(−)-epigallocatechin 3-O-gallate	0.16	6.7959
12	(−)-epigallocatechin (4β-8)-(−)-epicatechin-3-O-gallate	1.23	5.9101
13	(−)-epicatechin-3-O-gallate-(4β-8)-(−)-epigallocatechin3-O-gallate	0.93	6.0315
14	(−)-epigallocatechin-3-O-gallate-(4β-8)-(−)-epicatechin 3-O-gallate	0.69	6.1612
15	(−)-epiafzelechin 3-O-gallate	2.81	5.5513
16	(+)-catechin (4α-8) (−)-epigallocatechin	8.7	5.0605
17	(+)-catechin (4α-8) (−)-epigallocatechin 3-O-gallate	0.19	6.7212
18	(+)-gallocatechin (4α-8) (−)-epicatechin	3.28	5.4841
19	Oolongtheanin	0.25	6.6021
20	oolonghomobisflavan A	0.11	6.9586
21	mono-desgalloyl oolonghomobisflavan A	0.3	6.5229
22	di-desgalloyl oolonghomobisflavan B	2.29	5.6402
23	procyanidin B2	8.75	5.0580
24	procyanidin B3	3.28	5.4841
25	procyanidin B4	17.29	4.7622
26	prodelphinidin A2-3′-O-gallate	0.18	6.7447
27	prodelphinidin B2	3.24	5.4895
28	prodelphinidin B2- 3′-O-gallate	2.16	5.6655
29	prodelphinidin B2-3,3′-di-O-gallate	0.1	7.0000
30	prodelphinidin B4	6.85	5.1643
31	prodelphinidin B4-3-O-gallate	0.25	6.6021
32	prodelphinidin B5-3,3′-di-O-gallate	0.61	6.2147
33	theaflavin-3′-O-gallate	0.13	6.8861
34	theaflavin-3,3′-di-O-gallate	0.1	7.0000
35	theasinensin A	0.15	6.8239
36	theasinensin B	0.3	6.5229
37	theasinensin C	16.38	4.7857
