# Supplementary material for: Prediction and evaluation of the lipase inhibitory activities of tea polyphenols with 3D-QSAR models
Source: Sci Rep. 2016 Oct 3;6:34387. doi: 10.1038/srep34387 (PMC5046073; doi:10.1038/srep34387)
Supplement: Supplementary Information [file srep34387-s1.doc]

**Prediction and evaluation of the lipase inhibitory activities of tea polyphenols with 3D-QSAR models**

Yi-Fang Li^1,2,#^, Yi-Qun Chang^1,#^, Jie Deng^1,2,#^, Wei-Xi Li^2,3^, Jie Jian^1^, Jia-Suo Gao^1^, Xin Wan^1,2^, Hao Gao^1^, Hiroshi Kurihara^1,2^, Ping-Hua Sun^1,*^, Rong-Rong He^1,2,*^

^1^Guangdong Province Key Laboratory of Pharmacodynamic Constituents of TCM and New Drugs Research, College of Pharmacy, Jinan University, Guangzhou 510632, China

^2^Anti-stress Health Research Center, College of Pharmacy, Jinan University, Guangzhou 510632, China

^3^Yunnan University of Traditional Chinese Medicine, Kunming 650500, China

^*^Correspondence and requests for materials should be addressed to Rong-Rong He (rongronghe@jnu.edu.cn) and Ping-Hua Sun (pinghuasunny@163.com)

^#^These authors contributed to the work equally.

**Figure S1. Structure and atom numbers of DOTD**

DOTD

Amorphous powder. HR-ESI-MS: m/z 885.1450 (calcd for C_43_H_33_O_21_, 855.1509). ^1^H NMR (acetone-*d_6_*+D_2_O**,** 400 MHz), A,A’-rings: *δ*_H_ 5.76 (1H, d, *J* = 2.3 Hz), 5.86 (1H, d, *J* = 2.3 Hz), 6.00 (1H，d，*J* = 2.3 Hz), 6.04 (1H，d，*J* = 2.3 Hz) (6,6’,8,8’-H, overlapped); B,B’-rings: *δ*_H_ 6.87 (1H, s, H-6), 6.70 (1H, s, H-2’), 4.83 (1H, s, H-5’); C,C’-rings: *δ*_H_ 5.33 (1H, s, H-2), 5.45 (1H, m, H-3), 3.13 (1H, dd, J = 16.0, 4.4 Hz, H-4a), 2.89 (1H, dd, J = 16.0, 3.2 Hz, H-4b), 5.46 (1H, m, H-2’), 4.42 (1H, m, H-3’), 2.76 (1H, d, *J* = 12.0 Hz, H-4’a), 2.86 (1H, d, *J* = 12.0 Hz, H-4’b). Galloyl unit: 7.04 (s, 2,6-H). ^13^C NMR (acetone-*d_6_*+D_2_O**,** 100 MHz), A, A’-rings: *δ*_C_ 99.5 (C-4a), 99.6 (C-4’a), 95.9 and 96.8 (6,6’,8,8’-C, overlapped), 157.3 (C-5), 157.9 (C-5’), 157.7 (C-7), 158.0 (C-7’), 158.2 (8a, 8’a-C, overlapped); B,B’-rings: *δ*_C_ 131.3(C-1), 115.6(C-2), 148.9(C-3), 128.6(C-4), 147.9(C-5), 109.6(C-6), 156.7(C-1’), 125.3(C-2’), 200.2(C-3’), 94.5(C-4’), 54.3(C-5’); C,C’-rings: *δ*_C_ 78.5(C-2), 69.1 (C-3), 26.8 (C-4), 76.8 (C-2), 67.2 (C-3’), 27.1 (C-4’); Gallyoyl: *δ*_C_ 167.9 and 167.4 (-COO-), 121.6 and 121.3 (C-1), 110.5 (2,6-C, overlapped), 139.2 and 140.4 (C-4), 146.3 and 146.5 (3,5-C).
